# Pathological characteristics of esophageal cancer

**DOI:** 10.3892/ol.2014.2223

**Published:** 2014-06-04

**Authors:** HONG-YUN SHI, SHU-CHAI ZHU, WEN-BIN SHEN, MIAO-LING LIU

**Affiliations:** 1 Department of Radiation Oncology, Affiliated Hospital of Hebei University, Baoding, Hebei 071000, P.R. China; 2 Department of Radiation Oncology, The Fourth Hospital of Hebei Medical University, Shijiazhuang, Hebei 050017, P.R. China

**Keywords:** large pathological section, surgery, radiation, esophageal squamous cell carcinoma, pathological characteristics

## Abstract

The pathological characteristics of esophageal squamous cell carcinoma, which include regularly occurring multiple carcinogenic lesions (MLs), severe dysplasia (SD) and direct intramural infiltration (DI), were investigated using large pathological sections. A total of 52 esophageal cancer patients underwent surgical resection and were diagnosed with esophageal squamous cell carcinoma. Large sections of the surgical resection specimens were then made for pathological examination. The actual length of the carcinoma was calculated during surgery from the length determined microscopically. ML, SD and DI were identified during pathological examination of the large sections by microscope and were statistically analyzed. The lesion lengths obtained by the various inspection methods differed from each other. ML, SD and DI were identified in 15, 28 and 41 patients, respectively. Furthermore, a higher incidence of DI was observed in patients with lymphatic infiltration or those with a later stage of disease. ML, SD and DI were identified as characteristics of esophageal squamous cell carcinoma, and ML and DI were found to correlate with lymphatic infiltration.

## Introduction

Approximately 482,300 novel cases and 406,800 mortalities from esophageal cancer (EC) are reported annually worldwide (1). EC is the sixth most common cause of cancer mortality. The incidence of EC in China accounts for 50% of cases worldwide, and the incidence of EC in Henan province is >100/100,000 individuals. Although surgery, radiotherapy, chemotherapy and biologically targeted therapies continue to progress, the five-year survival rate for early EC patients undergoing surgical treatment is ~30%. The reason for the majority of failures is due to tumor recurrence (2–4). In addition, the use of radiotherapy has been a focus of debate. Therefore, the aim of the present study was to investigate the pathological occurrence of squamous cell carcinoma by investigating the pathological features of esophageal squamous cell carcinoma using large sections. This may guide doctors to determine the clinical target volume of radiotherapy, achieve a treatment for cancer and reduce the radiation doses to the normal tissues in order to improve a patient’s quality of life following treatment.

## Materials and methods

### Clinical data

A total of 52 EC patients who underwent radical resection in the Fourth Hospital of Hebei Medical University (Shijiazhuang, China) between 2011 and 2012 were included in the present study according to the following inclusion criteria: Pre-operative lesion length on esophagography of >3 cm; complete chest computed tomography (CT) scan, abdominal ultrasound and conventional biochemical test records; and pathological confirmation of squamous cell carcinoma. The 52 EC patients consisted of 34 males (65.4%) and 18 females (34.6%), with a mean age of 59 years (range, 26–73 years). The lesions were identified in the upper thorax of six cases, the middle thorax of 40 cases and the lower thorax of six cases. Esophageal imaging X-rays revealed a mean lesion length of 5.8±1.4 cm (range, 3–10 cm). Three cases were stage T2 and 49 cases were stage T3, 28 cases were stage N0 and 24 cases were stage N1, and 51 cases were stage M0 and one case was stage M1. In addition, 29 cases were stage II, 22 cases were stage III and one case was stage IV. TNM staging was determined according to the Union for International Cancer Control (5). Patients provided written informed consent. The study was approved by the ethics committee of The Fourth Hospital of Hebei Medical University.

### Detection methods and experimental procedure

The lesions and esophagus-related indicators were measured intraoperatively, and specimens were fixed for 24 h. The tumor length, invasion depth and the distances between the upper edge and the upper cutting edge of the tumor and between the lower edge and the lower cutting edge were measured without tension during surgery. The excised EC specimens were fixed with 10% formalin solution for 24 h, and the length of the specimens and invasion depths were measured. The types of tumor were identified and recorded.

To produce the large sections for pathological examination, 10% formaldehyde solution was used to fix specimens for >24 h, and two 0.3-cm wide tissue blocks were obtained from the fixed specimens. The tissue blocks contained the longest longitudinal section and both sides of the excised esophagus. Gauze (20 mesh) was used to produce the tissue boxes to avoid any distortion in the process of section production.

Following dehydration, embedding, sectioning, staining, dehydration transparency and cementing, the large pathological sections were completed.

**Figure 1:**
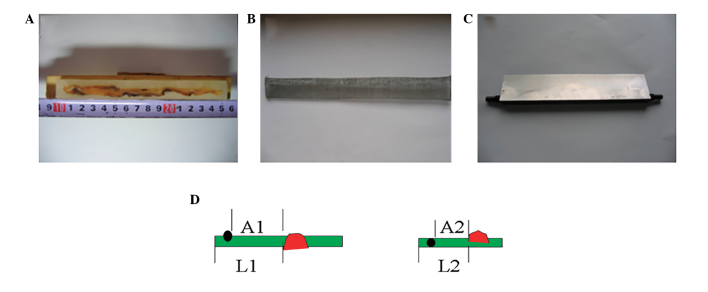
Equipment used to produce the large sections for pathological examination, and diagram of retraction during surgery.(A) Paraffin-embedded specimens of esophageal cancer, (B) a homemade tissue box and (C) a slicing knife. (D) Diagram of retraction during surgery (L1, normal esophagus above the esophageal tumor and A1, distance between ML and main tumor prior to surgery) and of the large sections (L2, normal esophagus above the esophageal tumor and A2, the distance between ML and main tumor following surgery). Red presents the main tumor and black the ML. L1/L2 = A1/A2. This equation allows the distance between the ML and the main tumor in the patient prior to the operation to be calculated. ML, multiple carcinogenic lesion.

For the diagnosis and measurement of the associated indicators, the sections were observed under a microscope to identify the occurrence of multiple carcinogenic lesions (MLs), severe dysplasia (SD), direct intramural infiltration (DI) (Fig. 1), lymph node metastasis, vascular invasion and lymphatic invasion.

The diagnostic criteria were as follows: ML, foci separated from the main tumor exhibiting characteristics of atypical hyperplasia carcinoma *in situ* or invasive carcinoma; SD, undifferentiated atypical cells accounting for >2/3 of the esophageal epithelial cell layer without breaking through the underlying membrane; and DI, normal mucosal epithelial lining of the esophagus with cancer submucosal infiltration or myometrial invasion.

**Figure 2:**
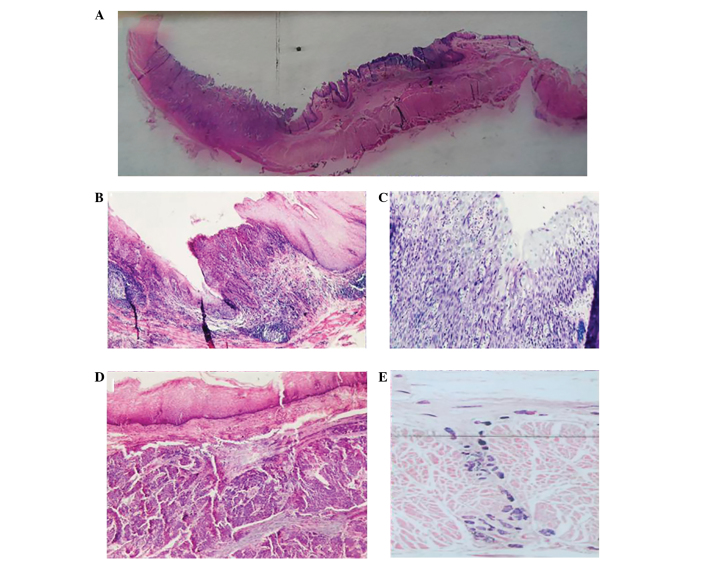
Large pathological section. (A) Unaided view and endoscopic views of the (B) multiple carcinogenic lesion (ML), (C) severe dysplasia (SD), (D) direct intramural infiltration (DI) and (E) lymphatic invasion.

The actual length of the carcinomas were calculated from the length determined microscopically, using the following equation: L1/L2 = A1/A2 where L1 is the length of the normal esophagus above the esophageal tumor and A1 is the distance between ML and main tumor prior to surgery. L2 is the normal esophagus above the esophageal tumor and A2 is the distance between ML and main tumor following surgery. Using this equation the distance between the ML and the main tumor in the patient prior to the operation may be calculated. All other actual lengths were calculated using the same method (Fig. 2).

### Statistical analysis

Statistical analyses were conducted using SPSS version 11.5 (SPSS, Inc., Chicago, IL, USA) and data are presented as the mean ± standard deviation. The means of the two groups were compared by t-test, and the means of multiple groups were compared by analysis of variance and χ^2^ test. Correlations between the pathological features were analyzed by the Pearson’s correlation test. P<0.05 was considered to indicate a statistically significant difference.

## Results

### Comparison of lesion lengths

The comparison between lesion lengths determined by esophagography, esophagoscopy and CT scan, and those measured during surgery are shown in Table I. The mean lesion length determined by esophagoscopy was 5.20±1.64 cm, which was significantly different (P=0.004) from the mean lesion length observed during surgery (5.83±1.41 cm). Esophageal stenosis, which inhibits esophagoscopy, may account for this. The lesion lengths determined by CT scan were the largest. This may be associated with the thickening of the esophageal lumen due to esophageal edema around the EC.

### Vascular tumor emboli and lymphatic invasion

Vascular tumor emboli were identified in six out of 52 patients, accounting for 11.5% of the total number of patients. Statistical analysis was not performed, as only six cases were identified. In addition, lymphatic invasion was observed in 36 out of 52 patients, accounting for 69.2% of the total number of patients.

### Distribution and factors affecting ML

A total of 28.8% of the patients (15 out of 52) were found to exhibit ML, with seven cases identified at the proximal end (upper) of the section, three cases at the distal end (lower) and five cases at both ends. The mean proximal distance between the ML and the main tumor, in addition to the length of the ML was 3.02±1.45 cm (largest length, 5.0 cm). In 95% of patients, a distance of <5.0 cm was exhibited, while 90% of patients exhibited a distance of <4.5 cm. The mean distal distance between the ML and the main tumor, in addition to the length of the ML was 2.60±2.44 cm (largest length, 7.5 cm). In 95% of patients, a distance of <7.5 cm was exhibited, while 90% of patients exhibited a distance <5.0 cm. No significant difference was identified between the mean proximal and mean distal distances (P=0.633; Table II).

The lesion length and invasion depth were reduced in the patients with ML compared with the patients without ML (P=0.039 and 0.028, respectively), however, no significant difference was identified in the tumor volume between the two groups (Table III). Lymphatic invasion was identified in 36 cases and of these, 14 were found to exhibit ML, accounting for 38.9%, whereas only one patient was identified with ML from the 16 patients without lymphatic invasion (6.3%). A statistically significant difference was identified between the two groups (P=0.036). However, no significant correlation was identified between the ML, type of primary tumor, lesion position, N stage and TNM stage (Table IV).

### Distribution and factors affecting SD

A total of 53.8% of patients (28 out of 52) were found to exhibit SD, with 11 cases identified at the proximal end of the section, 11 cases at the distal end and six cases at both ends. The mean proximal distance between the SD and the main tumor, in addition to the length of the SD was 2.45±1.30 cm. The mean distal distance between the SD and the main tumor, in addition to the length of the SD was 3.24±2.18 cm. No significant difference was identified between the mean proximal and mean distal distances (P=0.229; Table II). In addition, no significant correlation was identified between the lesion length, invasion depth and tumor volume and the SD (Table III), or between the SD and the type of primary tumor, lymphatic invasion, lesion position, N stage and TNM stage (Table IV).

### Distribution and factors affecting DI

A total of 78.8% of patients (41 out of 52) were found to exhibit DI, with 12 cases identified at the proximal end of the section, 10 cases at the distal end and 19 cases at both ends. The mean proximal distance between the DI and the main tumor, in addition to the length of the DI was 2.80±1.52 cm (largest length, 6.4 cm). The mean distal distance between the DI and the main tumor, in addition to the length of the DI was 2.02±1.51 cm (largest length, 6.0 cm). The mean proximal distance was significantly larger than the mean distal distance (P=0.049; Table II). In addition, the invasion depths were larger in patients with DI when compared with that of patients without DI (P=0.047). No significant correlation was identified between the lesion length, and tumor volume and the DI (Table III). However, a significant correlation was identified between lymphatic invasion and TNM stage (P=0.044) and the DI (P=0.05). No significant correlation was identified between the tumor volume and lesion position and the N stage (Table IV).

## Discussion

Two hypotheses exist with regard to EC patients with ML (6,7); one proposes that EC evolved from a monoclonal cell, whereas the other hypothesizes that EC has a polyclonal origin. The reported incidence of EC patients with ML is 0.8%–10.8% (8). In the current study, the incidence of ML was 28.8%, which is markedly higher than the reported incidence in the literature. This may be due to the large sections used for pathological examination, which provide more information with regard to the tumor. It was also revealed that smaller tumors were found to correlate with a higher incidence of ML. This indicated that under the effect of the same outside carcinogenic factors, a fragment of normal esophageal tissue may exhibit atypical hyperplasia in multiple sites. This atypical hyperplasia may develop from mild to moderate hyperplasia, severe hyperplasia, carcinoma *in situ* or invasive carcinoma. The small foci of these different sites gradually grow and fuse into visible tumors, which induce clinical symptoms, including swallowing difficulty and hiccups. The incidence of ML is decreased when these multicarcinoma *in situ* or microinvasive carcinomas merge with each other. This may explain why certain pathologists have identified >8 ML in early EC (9). In the current study, the proportion of ML in patients with lymphatic invasion was identified to be larger than that of patients without lymphatic invasion, indicating that ML occurs simultaneously under the same pathogenic factors. An additional possibility is that cancer cells have migrated along the longitudinal lymphatics and settled at a certain point, and proliferation started from there.

In the current study, the incidence of SD was 53.8%. Epidemiological studies have shown that the incidence of EC requires a continuous spectrum evolution, i.e., esophageal squamous epithelium, mild atypical hyperplasia, moderate atypical hyperplasia, SD, carcinoma *in situ* and invasive carcinoma (10,11). Much focus has been applied to SD, which exhibits undifferentiated atypical cells that account for >2/3 of the esophageal epithelial cell layer, but do not break through the underlying membrane. Carcinoma *in situ* refers to cancerized epithelial layers, which do not break through the basement membrane or infiltrate downward. Tao and Zong (12) analyzed EC specimens and found that the p53-positive and SD rates in carcinoma *in situ* were 98 and 79%, respectively (P>0.05). Although the specimens of the two groups exhibited different clinical stages, no differences in SD or p53 protein expression in carcinoma *in situ* were identified, indicating that SD has a carcinomic nature. Therefore, the current study also observed the occurrence of SD and summarized its characteristics. DI and lymphatic invasion have been identified in EC, which has the biological behavior of infiltrating along the esophageal mucosa or spreading to the upper esophagus in lesions of ≤10 cm in size (13). Compared with solid tumors from other areas, EC exhibits a longer diffusion distance, as the cancer cells infiltrate the esophageal wall and spread to the surrounding tissue. Notably, cancer cells can infiltrate along the lymphatics in the lower lamina propria, and occasionally cause nodular mucosal surface uplift. However, in the majority of cases, no evident abnormalities are identified and confirmation by microscope is required. This diffusion may be identified 5 or even >10 cm away from the primary tumor range, according to the published data. The majority of the literature supports the hypothesis that the diffusion distance to the upper end is larger than that to the lower end (13), which was also confirmed in the current study.

Although large sections for pathological study have a long production period, the entire tumor is included, as well as the proximal and distal resected tissues. The end results also have more integrity and continuity, and are excellent for investigating the associations between tumors and their surrounding tissues.

In conclusion, esophageal squamous cell carcinoma exhibits the characteristics of ML, SD and DI, and the occurrence of ML and SD has been associated with lymphatic invasion. In 95% of the subclinical lesions observed in the current study, the proximal distance was 5.0 cm and the distal distance was 7.5 cm, while in 90% of the subclinical lesions, the proximal distance was 4.7 cm and the distal distance was 5.0 cm. Further information may be obtained by future investigations of the pathological characteristics of EC, which could be used to identify a potential breakthrough in cancer research to relieve the suffering of patients, while reducing normal tissue damage and maximally improving quality of life.

## Figures and Tables

**Table I:** Comparison between tumor lengths measured during surgery and other methods.

Measuring method	Mean, cm	T-value	P-value
Surgery	5.83±1.41		
Esophagography	5.51±1.46	2.495	0.016
Esophagoscopy	5.20±1.64	3.056	0.004
CT scan	6.30±2.81	2.064	0.042

CT, computed tomography.

**Table II:** Distances between the subclinical lesion and the main tumor in addition to the subclinical lesion itself.

Subclinical lesion	Maximum, cm	Mean, cm^a^	95% CI, cm	90% CI, cm	T-value	P-value
ML						
Proximal end	5.0	3.02±1.45	≤5.0	≤4.5	0.486	0.633
Distal end	7.5	2.60±2.44	≤7.5	≤5.0		
SD						
Proximal end	5.0	2.45±1.30	≤4.5	≤3.8	1.232	0.229
Distal end	6.25	3.24±2.18	≤6.0	≤5.0		
DI						
Proximal end	6.4	2.80±1.52	≤5.0	≤4.7	2.012	0.049
Distal end	6.0	2.02±1.51	≤4.8	≤3.9		

a Data are presented as the mean ± standard deviation.

CI, confidence interval; ML, multiple carcinogenic lesion; SD, severe dysplasia; DI, direct intramural infiltration.

**Table III:** Correlation between the subclinical lesions and local tumor factors.

Subclinical lesion	Tumor length			Tumor invasion			Tumor volume		
		
	Mean, cm^a^	T-value	P-value	Mean, cm^a^	T-value	P-value	Mean, cm^3^^a^	T-value	P-value
ML									
Yes	4.46±1.15	2.125	0.039	2.95±0.53	2.262	0.028	26.83±11.84	0.929	0.357
No	5.49±1.73			3.35±0.59			33.27±10.59		
SD									
Yes	5.64±1.10	0.984	0.331	3.54±1.14	0.807	0.423	29.02±14.24	0.900	0.373
No	6.04±1.71			3.79±1.14			32.94±17.20		
DI									
Yes	5.80±1.50	0.216	0.830	3.77±1.13	2.040	0.047	30.03±16.38	0.708	0.482
No	5.91±1.04			3.00±0.89			33.81±12.75		

a Data are presented as the mean ± standard deviation.

ML, multiple carcinogenic lesion; SD, severe dysplasia; DI, direct intramural infiltration.

**Table IV:** Factors affecting the subclinical lesions.

	ML				SD				DI			
			
Factors	No, n	Yes, n	χ^2^	P-value	No, n	Yes, n	χ^2^	P-value	No, n	Yes, n	χ^2^	P-value
Lesion position			1.21	0.539			0.09	0.947			0.62	0.780
Upper esophagus	5	1			3	3			1	4		
Middle esophagus	25	11			16	20			6	31		
Lower esophagus	5	1			3	3			2	4		
Lymphatic invasion			4.15	0.038			0.18	0.675			4.12	0.043
Yes	20	12			15	18			5	30		
No	15	1			7	8			5	8		
N stage			3.42	0.057			0.43	0.691			1.82	0.183
N0	21	4			11	19			4	21		
N1	14	9			11	7			5	16		
TNM stage			2.09	0.135			0.14	0.718				
II	21	5			13	14			7	19	3.73	0.050
III + IV	14	8			9	12			2	20		

ML, multiple carcinogenic lesion; SD, severe dysplasia; DI, direct intramural infiltration.
